# Centromeric and pericentric transcription and transcripts: their intricate relationships, regulation, and functions

**DOI:** 10.1007/s00412-023-00801-x

**Published:** 2023-07-04

**Authors:** Jing Zhu, Qiao Guo, Minjun Choi, Zhoubin Liang, Karen Wing Yee Yuen

**Affiliations:** 1 School of Biological Sciences, The University of Hong Kong, Kadoorie Biological Sciences Building, Pokfulam Road, Hong Kong, China; 2 Institute of Molecular Physiology, Gaoke Innovation Center, Shenzhen Bay Laboratory, Guangming District, Guangqiao Road, Shenzhen, China

**Keywords:** Epigenetics, Centromere, Centromeric and pericentric transcription, Centromeric and pericentric RNAs, Chromatin environment, Non-coding RNA

## Abstract

Centromeres are no longer considered to be silent. Both centromeric and pericentric transcription have been discovered, and their RNA transcripts have been characterized and probed for functions in numerous monocentric model organisms recently. Here, we will discuss the challenges in centromere transcription studies due to the repetitive nature and sequence similarity in centromeric and pericentric regions. Various technological breakthroughs have helped to tackle these challenges and reveal unique features of the centromeres and pericentromeres. We will briefly introduce these techniques, including third-generation long-read DNA and RNA sequencing, protein-DNA and RNA–DNA interaction detection methods, and epigenomic and nucleosomal mapping techniques. Interestingly, some newly analyzed repeat-based holocentromeres also resemble the architecture and the transcription behavior of monocentromeres. We will summarize evidences that support the functions of the transcription process and stalling, and those that support the functions of the centromeric and pericentric RNAs. The processing of centromeric and pericentric RNAs into multiple variants and their diverse structures may also provide clues to their functions. How future studies may address the separation of functions of specific centromeric transcription steps, processing pathways, and the transcripts themselves will also be discussed.

## Introduction

### Centromere function

Historically, the centromere is often recognized as the constricted region that “holds” the X-shaped sister chromatids during cell divisions (Flemming 1880). Functionally, the centromere is the chromatin region where the kinetochore complex builds on, to connect chromosomes to microtubules emanated from the centrosomes. The centromere ensures that the sister chromatids and homologous chromosomes are separated equally to the daughter cells during mitosis and meiosis, respectively. Errors in chromosome segregation can cause chromosome breakages, or gains or losses of genetic materials (Potapova & Gorbsky 2017). The consequence is usually catastrophic for the cell and the whole organism, such as infertility, chromosomal abnormality disorders, or aberrant proliferation in cancers (Santaguida & Amon 2015; Smurova & De Wulf 2018).

### Centromere paradox and architectures

The conserved function of the centromere is contradicted with the diverse DNA sequences, sizes, and even architectures of the centromere across eukaryotes, and this phenomenon is called “centromere paradox” (Henikoff et al. 2001). There are two major architecture of centromeres: monocentromeres and holocentromeres (Wong et al. 2020). Monocentromeres, in which a single region of the chromosome is designated for the centromere function, are the most common architecture of centromeres in studied eukaryotes. Monocentromeres can be further subdivided into regional and point monocentromeres. The sizes of regional monocentromeres range from kilobases to megabases, and are mostly made up of AT-rich DNA (Baker & Rogers 2005; Barbosa et al. 2022), tandem repeats, called satellites, or transposable elements, as observed in many model organisms, including fission yeast, *Arabidopsis*, rice, flies, frogs, chicken, mice, tammar wallaby, and human cells (Hartley & O’Neill 2019; Shannon M McNulty & Sullivan 2018). The core centromere region of regional monocentromeres is flanked by pericentric, heterochromatic regions which are important for the cohesion of sister chromatids or homologous chromosomes during mitosis and meiotic reductional division, respectively. Meta-polycentromeres are monocentromeres that have distinct regions of centromeres believed to function together, forming an elongated primary constriction, as observed in *Pisum* and *Lathyrus* species (Schubert et al. 2020). Point monocentromeres refer to short centromeres in budding yeast, such as ~ 125 bp in *Saccharomyces cerevisiae*, which consists of 3 conserved elements (CDEI, II, and III), in which CDEII is AT-rich while CDEI and III are palindromic but are not repetitive (Clarke & Carbon 1980). Holocentromeres, in which the centromere is diffused along the length of the mitotic chromosomes, are observed in some plants, insects, and nematodes. Holocentromeres can be formed on the centromere-specific satellite family, called *Tyba*, and the centromeric retrotransposons (*CRRh*), as observed in *Rhynchospora pubera* (Marques et al. 2015), or on non-repetitive sequences, as observed in *Caenorhabditis elegans* (Gassmann et al. 2012; Talbert & Henikoff 2020). Notably, holocentromeres have evolved multiple times from their monocentromere ancestors independently in both animal and plant lineages (Escudero et al. 2016; Melters et al. 2012).

### Conserved epigenetic regulation of centromeres

Early mutational studies of centromeric DNA (Carbon & Clarke 1984; Cumberledge & Carbon 1987), centromere inactivation (Ishii et al. 2008; Thakur & Sanyal 2013), and the discoveries of neocentromeres, which are new centromeres formed on non-centromeric DNA sites (Scott & Sullivan 2014; Williams et al. 1998), have shown that centromeric DNA is not necessary or sufficient for centromere function, leading to the suggestion that most centromeres are epigenetically regulated (Allshire & Karpen 2008). One exception described is that of the point monocentromeres in *Saccharomyces cerevisiae*, as mutations in CDEIII cause centromere and kinetochore malfunction (Carbon & Clarke 1984; Ng et al. 1986). Many species, including *Saccharomyces cerevisiae*, contain a centromeric-specific histone H3 variant, CENP-A, that replaces canonical H3 at the core centromere, where kinetochore will assemble (Ali-Ahmad et al. 2020; Kixmoeller et al. 2020). Although there are exceptions, such as in silk moths *Bombyx mori* (Senaratne et al. 2021) and kinetoplastids (Ishii & Akiyoshi 2022), where there are no CENP-A, but they have histone-like CENP-T and unconventional KKT proteins, respectively. Understanding of the epigenetic regulation of centromeres begin with histone variant CENP-A, but later on also include unique centromeric histone modification combinations that are distinct from euchromatin and heterochromatin, DNA methylation pattern, centromeric and pericentric transcription, and centromeric and pericentric RNAs (cenRNAs and pericenRNAs) (Arunkumar and Melters 2020; Bergmann et al. 2012; Chan et al. 2012; Corless et al. 2020; Perea-Resa & Blower 2018; Saffery et al. 2003; Wong et al. 2007).

### Centromere is not an inert but an actively transcribed region, despite at a low level

The chromosome constriction site at the monocentromere was originally taken as a transcriptionally inert site. High level of transcription is incompatible with centromere function in budding yeast (Hill et al. 1987) and human artificial chromosome studies (Molina et al. 2016). However, in the last decade, more evidence from numerous studies in monocentromeres, even in the simplest point centromeres in *Saccharomyces cerevisiae* (Hedouin et al. 2022; Ling & Yuen 2019; Ohkuni & Kitagawa 2012), have shown that both the core centromere region and flanking pericentric regions are transcribed by RNA polymerase II (RNA Pol II) at a relatively low level and in a cell cycle-dependent manner (reviewed in Duda et al. 2017; Perea-Resa & Blower 2018; Smurova & De Wulf 2018). The functions of cenRNAs at the centromere, the inner centromere (the region between the sister chromatids), and the kinetochore have been reviewed thoroughly and recently (Corless et al. 2020). The relationship between cenRNA expression and CENP-A loading time in the cell cycle in different organisms has also been elaborately discussed (Perea-Resa et al. 2018).

Here, we will focus on the discussion of ongoing challenges and recent breakthroughs relevant to studying centromeric transcription, including the repetitive nature of centromeric DNA sequences, the difficulty to distinguish centromeric and pericentric sequences and their corresponding transcription in some species, and the difficulty in separating the function of the centromeric transcription process versus the centromeric RNA transcripts.

## Challenges

### DNA and thus RNA repetitiveness in many regional monocentromeres

The repetitive problem has been discussed in details (Corless et al. 2020). Mapping of RNAs to unique locations will rely on a complete, reliable genomic DNA reference. Recent studies, based on third-generation long-read DNA sequencing, such as Nanopore and PacBio Single-Molecule Real-Time (SMRT) sequencing, has assembled and polished individual human chromosomes Y and X from telomere-to-telomere using a bacterial artificial chromosome (BAC) vector or a complete hydatidiform mole (CHM)-derived haploid cell line, respectively (Jain et al. 2018; Miga et al. 2020; Nurk et al. 2022). Based on these frameworks, Miga’s group has also revealed the organization and evolutionary patterns of centromeric satellite arrays (Altemose et al. 2022a). Centromeric and pericentric satellites in human megabase-sized centromeres constitute 6% of the genome (Altemose et al. 2022a). Besides the major component, alpha-satellites, which comprised 171-bp monomers, other satellites (e.g., HSat2, HSat3, HSat1, beta-satellites) were also ordered. Different monomer subtypes (e.g., a, b, and c) are linked and repeated to form a higher order repeat (HOR) unit (e.g., abc). Identical HOR units then make up a large, homogenous HOR array (e.g., abc-abc-abc…) with thousands of HOR units. Centromeres from different chromosomes (e.g., Chr1, 5, 19) that are confounded previously can now be resolved based on distinct HOR arrays and chromosome-specific sequence variants, and verified by flow cytometry-sorted chromosomes. This pioneered work, together with direct RNA long-read sequencing (Jiang et al. 2019), opens up new avenues that will allow more precise mapping of long non-coding cenRNAs to the centromere.

In a detailed biochemical study by combining CUT&RUN (cleavage under targets and release using nuclease) of CENP-A/B/C and salt fractionation, Henikoff’s group has observed drastic CENP-A/B/C configuration differences on alpha-satellite dimers belonging to the same alpha-satellite subfamily that contain only 4–12% differences in sequence (Thakur et al. 2018). The configurations include a symmetric complex with equal CENP-A/B/C binding on both monomers of the dimer, or an asymmetric complex preferentially occupying only one monomer of the dimer (Thakur et al. 2018). This result suggests that even slight alpha-satellite sequence differences affect the binding behavior of the associated centromeric complex (Thakur et al. 2018). Their work may help us to correlate the CENP-A occupancy or chromatin states with the cenRNAs based on their sequences.

Alternatively, works from centromeres with unique sequences have circumvented the repetitive problem and provided evidence for centromere transcription. For example, some chicken and potato chromosomes contain repetitive centromere sequences while some do not (Gong et al. 2012; Shang et al. 2010). Interestingly, in chicken meiosis II prophase, centromeric transcription is only observed on the non-repetitive centromeres (Krasikova et al. 2012). In pathogenic yeast *Candida albicans*, each centromere has a unique central core (Sanyal et al. 2004). The non-heterochromatic pericentric regions of *C. albicans* contain either long terminal repeats (LTR), inverted repeats, or non-repetitive sequences (Freire-Benéitez et al. 2016). Such chromosome-specific centromere sequences and organization may facilitate the identification of cenRNA in the future. In *S. cerevisiae*, centromeric transcripts corresponding to each of the 16 chromosomes have been detected, but they are more highly expressed in S phase (Hedouin et al. 2022; Ling & Yuen 2019; Ohkuni & Kitagawa 2012). A targeted RNA isoform long-read sequencing (Iso-seq) by PacBio SMRT with probe-based enrichment has enabled the identification of many centromeric (and pericentric) RNA variants with strand-specificity and co- or post-transcriptional processing information, such as poly-adenylated tails (Hedouin et al. 2022). While the level of centromeric transcripts from each chromosome varies (Hedouin et al. 2022; Ling & Yuen 2019), conversion of all chromosomes’ centromeres to the same centromeric DNA sequence has suggested that the level of centromeric transcripts roughly correlates to the copy number of that specific centromeric DNA sequence in the genome, and they may function *in trans*, potentially in proximity to the centromere cluster (Jin et al. 1998; Ling & Yuen 2019).

### Distinguishing centromeric versus pericentric sequences

In humans, alpha-satellite sequences make up both the core centromeric and most of the flanking pericentric regions. It has long been difficult to differentiate between transcripts derived from these two regions due to their similarity. Analyses of long-read DNA sequencing and existing native (uncrosslinked) chromatin immunoprecipitation followed by DNA sequencing (N-ChIP-seq) and CUT&RUN datasets show that only a subset of human alpha-satellite HOR units bind CENP-A and assemble kinetochore proteins, and are known as the “active” array (340 kb to 4.8 Mb) (Altemose et al. 2022a). Flanking pericentric “inactive” arrays comprised more diverged alpha-satellites, other repeat families, and transposable elements. There is evidence of layered expansion, where new, distinct alpha-satellite repeat emerges and expands within the array to become the site of kinetochore assembly (the active array), whereas older repeats are displaced sideways symmetrically to become the inactive pericentric “layers.” However, there are also individual variations in terms of CENP-A localization on the newer or older repeats, suggesting epigenetic plasticity.

Identification of large structural rearrangements, transposable elements, or gene interspersion within the pericentric regions also help to distinguish among chromosomes (Altemose et al. 2022a), and may assist the annotation of transcribed cenRNAs or pericenRNAs. In the future, techniques that allow analyses of protein-DNA interactions with long-read information, such as DiMeLo-seq, will be useful to decipher the relationship between kinetochore function and underlying DNA sequences, individual variations, and evolutionary trends (Altemose et al. 2022b), and compare to the long RNA reads.

Besides the newly identified alpha-satellite HOR differences between human centromeric and pericentric regions, researchers have elucidated that the chromatin environment in these two regions vary significantly mostly by microscopy approaches. For instance, the chromatin at the core centromere typically contains histone modification marks associated with open chromatin or permissive transcription (Bergmann et al. 2012; Bergmann et al. 2011; Gopalakrishnan et al. 2009; Sullivan et al. 2004). CENP-A nucleosomes within the core centromere are interspersed with canonical histone H3 nucleosomes where H3 tails are modified with H3K4me1, H3K4me2, H3K36me2, and H3K36me3 (Table 1). These modifications are essential for CENP-A chaperone, HJURP, targeting, and CENP-A assembly (Bergmann et al. 2011; Duda et al. 2017). H3K4me2 is a modification associated with open but not active euchromatin (Sullivan et al. 2004; Soares et al. 2017). H3K36me2 is enriched downstream of transcription start sites (Weinberg et al. 2019) and can recruit histone deacetylases (HDAC) enzymes (Li et al. 2009). The core centromere also has reduced CpG methylation, called centromere dip region (CDR), consistent with its open chromatin (Altemose et al. 2022a).

**Table 1:** Transcription-related “histone code” on the core centromere and pericentromere

Histone modification	Underlying transcriptional activity ( +) or silencing ( −)	Known function in transcription	Location (core centromere/pericentromere)	Evidence and possible mechanism of work on the centromere
H3K36me3	+	• Is often associated with gene bodies, especially enriched in exons (Schwartz et al. 2009) • Binds to histone deacetylase (HDAC) which deacetylates histones and prevent run-away transcription (Carrozza et al. 2005) • Is associated with both facultative and constitutive heterochromatin (Chantalat et al. 2011)	Core centromere	• Marks core centromere region (Gopalakrishnan et al. 2009; Sullivan et al. 2004) • Thought to promote RNA Pol II activity and are essential for HJURP targeting and CENP-A assembly
H3K36me2	+	• Is enriched downstream of transcription start sites (Weinberg et al. 2019) • Can recruit HDAC enzymes (Li et al. 2009)	Core centromere	
H4K20me1	+	• Is associated with transcriptional activation (Wang et al. 2008) • The most highly transcribed group of genes tend to have H4K20me1 present at active promoters (Wang et al. 2008)	Core centromere	• Highly enriched in human and chicken centromere and are required for normal kinetochore function (Hori et al. 2014) • Possibly functions as a mark of CENP-A chromatin maturation (Nam & Bartel 2012)
H3K4me2	+	• Is a modification associated with open but not active euchromatin (Soares et al. 2017; Sullivan et al. 2004)	Core centromere	• Found in human and *Drosophila* centromere, but not in rice or chicken centromere (Mravinac et al. 2009; Sullivan et al. 2004) • Required for HAC formation (Bergmann et al. 2011)
H3K4me1	+	• Is often associated with gene enhancers (Rada-Iglesias 2018)	Core centromere	• Thought to provide an open chromatin for CENP-A incorporation and recruitment of other centromere and kinetochore proteins
H3K9ac	+	• Is highly correlated with active promoters (Karmodiya, Krebs, Oulad-Abdelghani, Kimura, & Tora, 2012) • Can turn genes on (Karmodiya et al. 2012)	Core centromere	• Is shown to be required for neocentromere formation in *C. elegans* (Zhu et al. 2018a) • Promotes neocentromere formation in HAC (Bergmann et al. 2012; Ohzeki et al. 2012) • Provides open chromatin for ectopic CENP-A incorporation and recruitment of other centromere and kinetochore proteins
H4K5ac	+	• It is enriched at the transcription start site (TSS) and along gene bodies (Lennartsson & Ekwall 2009)	Core centromere	• In chicken cells, H4K5ac and H4K12ac, which correlate with transcribed chromatin, are found enriched at centromere core and are essential for CENP-A deposition (Shang et al. 2016)
H4K12ac	+	• Is localized to the promoter (Wang et al. 2008)	Core centromere	
H3K27me3	−	• Correlates with transcriptional repression (Ferrari et al. 2014) • Contribute to compaction of the chromatin (Rougeulle et al. 2004)	Pericentromere	• Marks pericentromere region (Kundaje et al. 2015) • Is predicted to help maintain neocentromere once it was formed, and is positively correlated with CENP-A in *C. elegans* (Gassmann et al. 2012)
H3K27me2	-	• Has a broad distribution and a role in silencing non-cell-type-specific enhancers (Ferrari et al. 2014)	pericentromere	• Marks pericentromere region (Kundaje et al. 2015)
H3K9me3	−	• Is found more often at silenced genes (Barski et al. 2007) • Binds heterochromatin protein 1 (HP1) at constitutive heterochromatin (Lehnertz et al. 2003), which is responsible for transcriptional repression and the actual formation and maintenance of heterochromatin	Core centromere and pericentromere	• Absent in human and *Drosophila* centromere (Mravinac et al. 2009; Sullivan et al. 2004) • It has also been shown to be compatible with the centromeric nucleosomes (Bergmann et al. 2012; Ribeiro et al. 2010) • Antagonize H3K9ac, which is required for neocentromere formation in *C. elegans* (Zhu et al. 2018a) • Induces a closed chromatin environment, which may prevent ectopic CENP-A incorporation
H3K9me2	-	• Is found more often at silenced genes (Barski et al. 2007) • Is a characteristic mark of the inactivated X chromosome (Xi) (Rougeulle et al. 2004)	pericentromere	• Marks pericentromere region (Sullivan et al. 2004) • Induces a closed chromatin environment, which may prevent ectopic CENP-A incorporation

On human artificial chromosomes (HACs), core centromere transcription can promote H3K9ac accumulation, which in turn can prevent heterochromatin formation at this region (Molina et al. 2016). Yet, on the same site, H3K9me3, typically thought to be associated with transcriptional repression, has also been shown to be compatible with the centromeric nucleosomes (Bergmann et al. 2012; Ribeiro et al. 2010). However, in general, the core centromere lacks clear marks for heterochromatin, such as H3K9me2 (Sullivan et al. 2004). A balance between euchromatin and heterochromatin characteristics at the core centromeric region is required for maintaining a low level of centromere transcription, maintaining the centromere identity and forming an active kinetochore (Sullivan et al. 2004). On the other hand, the surrounding pericentric regions are marked by histone modifications typically associated with transcriptional silencing, such as H3K9me2, H3K9me3, H3K27me2, and H3K27me3 (Table 1) (Gopalakrishnan et al. 2009; Kundaje et al. 2015).

At the core centromere, histone H4 tails are also modified. In chicken cells, H4K5ac and H4K12ac, which correlate with transcribed chromatin, are found to be enriched at the core centromere CENP-A pre-nucleosomes and are essential for CENP-A deposition (Shang et al. 2016). H4K20me, which is associated with transcriptional activation, is found in chicken and human CENP-A nucleosomes and is a prerequisite for kinetochore assembly (Bergmann et al. 2011; Hori et al. 2014; Sullivan et al. 2004).

For organisms in which the centromeric and pericentric DNA sequences can be differentiated, the transcripts levels and functions can be separately analyzed. For example, in *S. pombe*, the core centromere CENP-A/Cnp1-containing region consists of the non-repetitive central core (*cnt*) and flanking innermost repeats (*imr*). The core centromere is separated from the pericentric outer repeats (*otr*) by tRNA arrays (Dawe 2003). There is evidence of *cnt*, *imr*, and *otr* expressions by RNA Pol II at different lengths, levels, and functions. The tRNA genes at the barrier are also expressed by RNA Pol III. The pericentric RNAs are processed into small interfering RNAs (siRNAs) importantly for heterochromatin establishment, which is in turn required for de novo CENP-A establishment on introduced centromere-containing plasmid (Folco et al. 2008). Interestingly, tethering of histone H3 lysine 9 methyltransferase Clr4 to generate synthetic heterochromatin can bypass the need of RNA interference (RNAi) and the presence of outer repeats for centromere establishment (Chan & Wong 2012; Kagansky et al. 2009).

*S. cerevisiae* does not have heterochromatic pericentric regions, but the pericentric regions are characterized by H2A.Z-containing nucleosomes, in which this H2A variant is known to regulate sister chromatid cohesion and gene expression (Giaimo et al. 2019; Sharma et al. 2013). Indeed, centromeric transcription is mainly readthrough of pericentromeric regions with highly heterogeneous transcriptional start sites (TSS) but rarely span the entire centromere (Hedouin et al. 2022), consistent with RNA Pol II stalling in CDEI and CDEIII (Candelli et al. 2018).

In *Drosophila*, previous ChIP-seq revealed that CENP-A associates with simple satellites (Talbert et al. 2018). Yet, recent chromatin fibers, long-read DNA sequencing, and CENP-A/CID ChIP-seq that mapped to a heterochromatin-enriched genome assembly have revealed that CENP-A chromatin is formed on retroelement *G2/Jockey-3*, which is the only element shared among all chromosomes’ centromeres (Chang et al. 2019). This core centromere is then flanked by large arrays of satellite repeats (Chang et al. 2019). Low levels of *G2/Jockey-3* transcription has been detected from some chromosomes’ centromeres but not all (Chang et al. 2019). Satellite RNA has also been shown to bind CENP-C and help to localize CENP-C to the centromere (Rošić et al. 2014). The refined centromere and pericentromere organization will facilitate the separation of transcription in these two regions.

Analyses of an evolutionary neocentromere (ENC), which is formed on non-centromeric DNA sequences and has been propagated across multiple generations in humans, show that the new CENP-A-defined core neocentromere becomes enriched in active epigenetic marks, RNA Pol II, and negatively supercoiled DNA, consistent with active transcription. In contrast, there is a spreading of repressive epigenetic marks, e.g., H3K27me3, to the surrounding regions (Naughton et al. 2022). The authors suggested that transcription disrupts chromatin to provide a foundation for kinetochore formation, while compact pericentric heterochromatin generates mechanical rigidity (Naughton et al. 2022).

Besides centromere and pericentric sequence and epigenetic differences, a recent study has investigated the nucleosomal patterns in different satellite sequences by a newly developed method called single-molecule adenine methylated oligonucleosome sequencing assay (SAMOSA), which combines non-specific adenine (m6dA) methyltransferase footprinting and single-molecule, real-time PacBio DNA sequencing to natively and non-destructively measure nucleosome positions, regularity, and nucleosome repeat lengths (NRLs) on individual chromatin fibers (500 b–2 kb) (Abdulhay et al. 2020). Interestingly, in human K562 cells, H3K9me3-decorated alpha- and beta-satellite sequences are enriched for both the expected regular and the unexpected irregular fibers (Abdulhay et al. 2020). The results show that heterochromatic nucleosome conformations can be both irregular and heterogenous. On the other hand, H3K9me3-free gamma-satellite is only enriched for chromatin fibers with regular long NRLs (Abdulhay et al. 2020). NRLs may specify the ability of heterochromatic nucleosomal arrays to phase separate (Gibson et al. 2019). With future optimization of digestion conditions, SAMOSA could be applicable to longer arrays, enabling kilobase-domain-scale study of single-molecule oligonucleosome patterning. In addition, multiple biochemical or epigenetic signals may be detected on the same single molecules, providing a “multi-omic” third-generation sequencing platform (Abdulhay et al. 2020).

### Does centromere transcription occur in holocentromeres?

A missing piece in the centromere transcription field is whether it also happens in meta-polycentromeres and holocentromeres. For repeat-based holocentromeres, recent PacBio HiFi long-read sequencing, ChIP-seq, and high‐throughput chromosome conformation capture (Hi-C) on three holocentric sedge *Rhynchospora* species and their closest monocentric relative, the rush *Juncus effusus*, have enabled us to compare their centromere and epigenome organization and 3D genome architecture (Hofstatter et al. 2022). CENP-A/centromeric histone H3 (CenH3) has the highest enrichment in *Tyba* repeats and a lower enrichment in *CRRh* throughout the entire *R. pubera* and *Rhynchospora breviuscula* genomes (Hofstatter et al. 2022). The density of the *Tyba* arrays decreases with chromosome size, and there is a high frequency of dyad symmetries in the *Tyba* consensus sequences (Hofstatter et al. 2022). In *R*. *pubera*, the CENP-A-binding *Tyba* satellites are transcriptionally active in all tissues, and these *Tyba* satellites and *CRRh* are inserted into transcriptionally active gene-containing chromatin regions as genome-wide interspersed arrays (Marques et al. 2015). Holocentric sedges have more intrachromosomal interactions than its monocentric relative due to the lack of centromere clustering (Hofstatter et al. 2022). These newly characterized repeat-based holocentromeres seem to have similarities with many regional centromeres. There is a slight enrichment of H3K9me2 flanking CENP-A domains relative to the core CENP-A region, mimicking the pericentromeric chromatin composition in monocentromeres (Hofstatter et al. 2022). Further studies will elucidate whether the *Tyba* and *CRRh* transcripts behave like cenRNA and pericenRNA, respectively.

However, in non-repeat-based holocentromeres, there is so far no concrete evidence of holocentromeric transcription. In nematode *C. elegans* (Gassmann et al. 2012) and lepidopteran *Bombyx mori* (Senaratne et al. 2021), CENP-A/HCP-3 or CENP-T localization has an anti-correlation with active transcribed regions. In moth tissue culture cells, hormone induction of transcription results in CENP-T loss in the regions, suggesting that transcription can exclude holocentromere locations (Senaratne et al. 2021). CENP-A in *C. elegans* embryos is also anti-correlated with not only actively transcribed genes in embryos, but also some germline-transcribed regions (Gassmann et al. 2012). Histone H3K36 methyltransferase *met-1* mutant causes ectopic H3K36me3 localization and exclusion of CENP-A, but not necessary an increase in active RNA Pol II localization at those sites (Gassmann et al. 2012). Argonaute CSR-1 is involved in a small RNA pathway that regulates germline gene expression (Claycomb et al. 2009). The germline target gene loci of CSR-1 anti-correlates with CENP-A localization (Gassmann et al. 2012). Disruption of CSR-1 pathway increases the level of CENP-A on chromatin (Wong & Yuen 2022). These results suggest that active or past gene transcription, histone modification, and 22G-sRNA may restrict CENP-A localization, which is consistent with the incompatibility of high gene expression with CENP-A in *C. albicans* and *S. cerevisiae* (Hill et al. 1987; Ketel et al. 2009).

On a *C. elegans* artificial chromosome (AC) with almost no genes, the CENP-A domain intervals and average domain size reduce, suggesting that more non-expressed regions are permissible holocentromere regions for CENP-A to locate (Lin et al. 2021). By imaging of artificial chromosomes, we found that inhibition of RNA Pol II-mediated transcription causes delayed de novo centromere establishment on newly formed ACs in holocentric *C. elegans* early embryos (Zhu et al. 2018a). However, we did not explore whether cenRNAs exist, or distinguish between the role of the act of transcription versus the cenRNA products during centromere establishment. So far, existing transcriptome analyses have detected many lowly abundant, long non-coding transcripts (Akay et al. 2019; Nam & Bartel 2012), but not directly related to holocentromeres. It will be interesting to learn whether holocentric transcription and cenRNAs exist, and their relationships with the centromere function, respectively.

### Differentiating the function of the act of transcription versus transcripts

RNA Pol II is responsible for centromere transcription in many species analyzed, as shown by the effects of using specific drugs that differentially inhibit RNA polymerase I, II, or III’s activity (Chan et al. 2012). Recently, immunofluorescence experiments have shown that RNA Pol II Serine 2 phosphorylation of the C-terminal domain (CTD), which correlates with the active transcription elongation, is present at fly, frog, and human centromeres during mitosis (Blower 2016; Chan et al. 2012; Molina et al. 2016; Rošić et al. 2014). The passage of RNA Pol II in centromere transcription will bring two effects: it can trigger a chromatin remodeling event and produce a centromeric RNA. So, although the loading of CENP-A at centromeres has been associated extensively with the transcription process in different organisms (Choi et al. 2011; Grenfell et al. 2016; Quénet & Dalal 2014), the underlying molecular mechanism is only beginning to be unveiled, which may assist us to separate the two effects of transcription.

#### Function of centromeric transcription

**Fig. 1:**
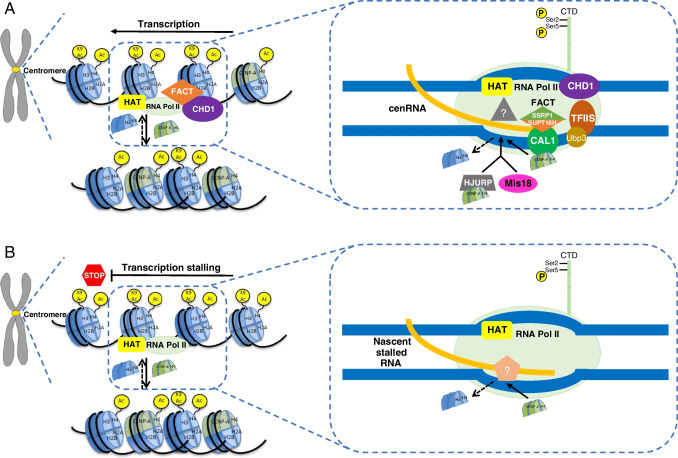
A schematic diagram outlining how centromeric transcription and stalling can regulate centromere CENP-A loading. **A** The chromatin environment changes and the “transcription bubble” generated by RNA Pol II passage favors the incorporation of CENP-A nucleosomes at centromere regions. Transcription-associated chromatin remodeling factors, including FACT complex and CHD1, and CENP-A chaperone such as CAL1 or HJURP together with MIS18 have been shown to be important for CENP-A deposition during the centromeric transcription. RNA Pol II phosphorylated at Ser2 of the C-terminal domain (CTD) is present at centromeres, indicating active transcription elongation through the action of RNA Pol II. The nucleosome destabilization activity of FACT complex, which consists of SSRP1 and SUPT16H subunits, could promote RNA Pol II elongation through the compact chromatin, while RNA Pol II transcription could drive further loosening at the centromere domain. FACT has been shown to interact with the CENP-A protein by interacting with CAL1, the CENP-A loading factor in *Drosophila*. FACT destabilizes H3 nucleosomes in order to promote CENP-A loading. RNA Pol II transcription could also recruit HAT complexes to the kinetochore to generate an acetylated chromatin environment, which has been shown to be favorable for CENP-A loading. Unknown factors involved in transcription elongation may also facilitate CENP-A deposition. **B** RNA Pol II stalling allows de novo establishment of CENP-A chromatin. Serine 2 in the CTD heptad repeat of RNA Pol II is phosphorylated in elongating RNA Pol II, and this RNA Pol II becomes ubiquitylated upon stalling. In *S. pombe*, newly introduced plasmid with the core centromere region will lead to transient stalling of RNA Pol II, but it can be efficiently cleared with the aid of factors such as TFIIS and Ubp3 (Kulish et al. 2001; Kvint et al. 2008; Martinez-Rucobo & Cramer 2013). TFIIS promotes transcriptional elongation by cleaving nascent transcripts in the context of stalled RNA Pol II. Mutants that lack Ubp3 or TFIIS compromise the restarting process of stalled RNA Pol II, resulting in the accumulation of stalled RNA Pol II complexes, prolonged stalling, and leading to recruitment of unknown factors that promote CENP-A deposition. The RNA Pol II stalling environment causes H3 nucleosomes to be efficiently evicted. CENP-A N-terminal tail lacks the conserved lysine residues of H3 (e.g., K9), and thus does not have H3K9ac-like modification that aids transcription. Therefore, CENP-A nucleosomes are likely to present a greater barrier to transcription than H3 nucleosomes. Thus, once CENP-A nucleosomes are loaded, it might exacerbate the transcriptional stalling, creating conditions permissive for recruitment of more CENP-A in a self-perpetuating way

Several previous studies have identified RNA Pol II-associated proteins and transcription-associated chromatin remodeling factors at the centromere. In eukaryotes, Facilitates Chromatin Transcription (FACT), a two-subunit complex containing SSRP1 and SUPT16H, is one of the major regulators of RNA Pol II transcriptional activity. FACT can be retained on DNA while RNA Pol II traverses pass FACT (Belotserkovskaya et al. 2003). FACT can promote RNA Pol II elongation by destabilizing or later stimulating formation of nucleosomes (Formosa 2012). Interestingly, both subunits of FACT interact with the CENP-A in different organisms (Barth et al. 2014; Foltz et al. 2006) and collaborate in transcription-coupled loading of CENP-A at the core centromere domain (Fig. 1A), by interacting with Chromosome Alignment Defect 1 (CAL1), the CENP-A/CID chaperone, in *Drosophila* (Chen et al. 2015). At ectopic centromeres in *Drosophila*, CAL1 also recruits FACT and RNA Pol II, which are essential for de novo CENP-A deposition (Chen et al. 2015). Immunostaining of FACT’s two subunits was the strongest at the kinetochore on mitotic chromosomes, at the same time that CENP-A normally loads in *Drosophila* tissue culture cells (Mellone et al. 2011). A more recent study using *Drosophila* tissue culture cells further proposed a two-step model for the recruitment and chromatin loading of CENP-A: at the beginning, CAL1-interacting CENP-A is recruited to the centromere domain in a manner that is independent of transcription and CENP-A is kept loosely associated with centromeric chromatin, then RNA Pol II passage facilitates the exchange of H3 nucleosomes to stably incorporate CENP-A into nucleosomes at the centromere (Bobkov et al. 2018). However, another recent study in *Drosophila* early embryogenesis has shown that RNA Pol II-mediated transcription is not necessary for the recruitment of CENP-A loading on the centromere (Ghosh & Lehner 2022). These contrasting results imply heterogeneity in the requirement of centromere transcription, which may depend on developmental stages or cell types.

A synthetic biology approach tethers different histone modifiers to the alphoid^tetO^ centromere on a human artificial chromosome (HAC) to manipulate histone modifications, and thus transcription, at the centromere (Molina et al. 2016). H3K9ac-associated but not H4K12ac-associated transcription rescues the loss of H3K4me2 and centromeric transcription (Molina et al. 2016). Thus, the authors proposed that both mitotic centromeric transcription and H3K9ac can generate an appropriate epigenetic landscape to destabilize H3 nucleosomes and promote CENP-A loading (Molina et al. 2016). In chicken B cell line DT-40, FACT localizes to the centromere in a CENP-H-dependent manner and associates with chromatin remodeler CHD1 to facilitate CENP-A loading (Okada et al. 2009), raising the possibility that CENP-A chaperone HJURP may act through a FACT-dependent mechanism similarly to CAL1 in *Drosophila*. On the other hand, in budding yeast *S. cerevisiae*, FACT functions in correcting ectopic CENP-A loading by binding with a E3-ubiquitin ligase and triggering the proteasome-mediated degradation of ectopic CENP-A (Deyter et al. 2014).

Besides transcription elongation, transcriptional stalling within the core centromere region has been suggested to promote de novo establishment of CENP-A chromatin in fission yeast (Fig. 1B) (Catania et al. 2015). The central core domain (*cnt*) of *S. pombe* contains numerous promoters and transcriptional start sites (Catania et al. 2015). The abilities of different parts of the *cnt* to assemble CENP-A chromatin were not equivalent. Surprisingly, the parts with lower transcriptional activity (high RNA Pol II level but low transcript levels) were more effective in recruiting CENP-A than those with higher activity. Mutants that are defective in restarting stalled RNA Pol II indeed increase CENP-A loading, suggesting that persistent RNA Pol II stalling creates a favorable chromatin environment for CENP-A loading, perhaps through dislodging H3 nucleosomes without eviction of CENP-A or providing a longer time for CENP-A recruitment (Catania et al. 2015). An in vitro experiment has also shown that CENP-A nucleosomes present a greater barrier to transcription than H3 nucleosomes (Shandilya et al. 2014), and CENP-A-induced stalling may potentially promote their own recruitment in a positive-feedback loop.

To further decipher which step during transcription may be important to facilitate CENP-A and kinetochore assembly, Heald’s group has used different drug treatments to inhibit specific stages of transcription in *Xenopus*. When transcription initiation is inhibited by triptolide or when RNA splicing process is inhibited by ISGN, a reduced level of CENP-A and kinetochore protein NDC-80 is observed (Grenfell et al. 2016). Yet when transcription elongation is inhibited by α-amanitin, no CENP-A incorporation effect is observed (Grenfell et al. 2016). These results may indicate that some transcription initiation or splicing factors are specifically involved in CENP-A recruitment.

From all these studies, the act of centromeric transcription and stalling, accompanied by the transcription-associated chromatin remodeling, play important roles in the establishment and maintenance of centromeric chromatin, particularly the stable incorporation of CENP-A into chromatin. However, to definitely conclude that the function solely comes from the transcription process and not from the cenRNAs will need more evidence to distinguish the two effects.

#### Structures and functions of cenRNA

In general, non-coding RNA can function as a guide to recruit proteins to DNA or chromatin, a scaffold to assemble ribonucleoprotein complexes, a decoy which bind and sequester proteins thereby inhibiting their normal functions, or an inter-cellular signaling molecule (Corless et al. 2020; Hezroni et al. 2015). There appears to be relatively more studies on manipulating the levels of cenRNAs than studies on the centromere transcription process. Centromere-derived RNAs have been proposed to play a critical role in proper centromere formation and function. Specifically, overexpression or knockdown of cenRNAs reduces CENP-A and CENP-C levels at the centromere (Ling & Yuen 2019; Liu et al. 2015; Rošić et al. 2014). Yet, the lack of cenRNA sequence conservation prompts us to speculate if cenRNAs have any conserved secondary and tertiary structures, which may enable them to recruit centromeric protein to the centromere.

Centromeric transcription often occurs in both sense and anti-sense strands (Ferri et al. 2009; Ling & Yuen 2019; Topp et al. 2004). Both long (> 200 nt) and short non-coding cenRNA and pericenRNA have been catalogued in previous reviews (Arunkumar and Melters 2020; Chan et al. 2012; Chan & Wong 2012). cenRNA structure has been proposed to be double-stranded (ds) RNA, single-stranded (ss) RNA, or a DNA-RNA hybrid with the complementary DNA sequence at the centromere (R-loops). Here, we describe evidence supporting different types of cenRNA structures (Table 2).

**Table 2:** Different structures of cenRNAs in different species

Structure	Species	Cell cycle	Functions and interacting proteins	Reference
Single-stranded RNA	Maize	Throughout cell cycle	Transcripts from centromeric retrotransposons and satellite repeats are bound to CENPA/CenH3 CENP-C binding to the centromeric DNA requires single-stranded centromeric transcripts	Du et al. (2010) and Topp et al. (2004)
	Human HeLa cells	Metaphase	Alpha-satellite derived RNAs play crucial role in CENP-C1, INCENP, and Survivin assembly to the centromere during metaphase	Wong et al. (2007)
Double-stranded RNA	Mouse	Mitosis	Accumulation of minor satellite transcripts leads to defective localization of centromeric proteins including Aurora-B and HP1	Ferri et al. (2009)
R-loops	Maize	Interphase	Circular RNAs at the centromeric regions forms R-loops and promotes the formation of chromatin loops. CENP-A/CenH3 localization is defective with decreased level of circular RNAs and chromatin loops	Liu et al. (2021) and Liu et al. (2020)
	Mouse	G1 phase	DNA-RNA hybrid facilitates homologous recombination repair and promote centromeric integrity	Yilmaz et al. (2021)
	*Schizosaccharomyces pombe*	S phase	DNA-RNA hybrids formed from heterochromatin-derived ncRNAs play significant role in RNAi-directed heterochromatin assembly	Nakama et al. (2012)
R-loops (negative effects)	*Saccharomyces cerevisiae*	S phase	R-loop accumulation at centromere leads to defective kinetochore biorientation and chromosome instability	Mishra et al. (2021)
	Human	Interphase	R-loop accumulation at centromeric alpha-satellite leads to mislocalization of CENP-A	Racca et al. (2021)

### Different structures of cenRNAs

#### Double-stranded (ds) cenRNA

Mouse minor satellites from the core centromere are transcribed and the transcripts are sensitive to RNase III, which cleaves dsRNA (Bouzinba-Segard et al. 2006). Tammar Wallaby’s Kangaroo Endogenous Retrovirus (KERV-1) exists as both dsRNA and ssRNA, which are important for CENP-B localization and pericentric heterochromatin maintenance (Carone et al. 2009). In *Schizosaccharomyces pombe*, ribonuclease Dicer, which cleaves pericentric *otr* dsRNA to 21–25-nt small interfering RNA (siRNA), and the RNA-induced initiation of transcriptional gene silencing (RITS) complex are important for CENP-A/Cnp1 establishment (Folco et al. 2008).

#### Single-stranded (ss) cenRNA

In maize, centromere retrotransponsons (CRM) and satellite repeats (CentC) are actively transcribed in both strands, and cenRNAs (40–250 nt) interact with CENP-A (Topp et al. 2004). However, there is no detection of centromere-derived small interfering RNAs (siRNAs) processed by the RNAi pathway. These cenRNAs are also sensitive to RNase A, suggesting that they maintain single-stranded organization (Topp et al. 2004). Single-stranded RNAs are highly unstable compared to double-stranded RNA (Zhang et al. 2021). However, RNA–protein interaction may stabilize the ss cenRNA. Further in vitro study has demonstrated that single-stranded maize cenRNA (44 nt) is essential for CENP-C exon duplication region to maintain DNA binding, and such cenRNA-CENP-C interaction is required for proper CENP-C centromere localization (Topp et al. 2004). In human HeLa cell line, alpha-satellite cenRNA, CENP-C1, and INCENP localize to the nucleolus in interphase in an RNA polymerase I-dependent manner, and this localization is also sensitive to RNase A (Wong et al. 2007). This suggests that human cenRNA also interacts as single-stranded structure with kinetochore or inner centromere protein.

In the case of transcription in the point centromere of *Saccharomyces cerevisiae*, after the introduction of RNAi machinery (but without adding the hairpin RNA against cenRNA), the level of cenRNAs is not affected, suggesting that endogenous cenRNAs probably do not exist as dsRNA (Ling & Yuen 2019). However, there is no direct evidence of ssRNA in budding yeast so far.

#### R-loop

Since cenRNAs are highly complementary with the template centromeric DNA, cenRNAs can potentially bind and form a R-loop structure consisting of a three-stranded DNA-RNA hybrid, with one strand of RNA bound to a single DNA strand, and a displaced single-stranded DNA. According to different studies, R-loops have been reported to be associated with gene transcription (Fang et al. 2019; Ling & Yuen 2019), DNA replication initiation (Yu et al. 2003), DNA damage response (Hamperl et al. 2017), DNA repair (Lu et al. 2018), and genome instability (Chedin & Benham 2020). The formation of DNA-RNA hybrids is also an important way to target the RNA to the local chromatin in a sequence-specific manner (Maldonado et al. 2019).

Generally, R-loops are formed with G-rich clusters during transcription (Allison & Wang 2019). However, the centromeric regions are often enriched with AT (Altemose et al. 2022a; Baker & Rogers 2005), which is not favorable for elongation and the formation of the R-loops. Additionally, negative supercoiling facilitates R-loop formation (Stolz et al. 2019). In *S. cerevisiae*, CENP-A/CSE4 induces positive supercoiling at the centromere (Furuyama & Henikoff 2009), which is unfavorable for R-loop formation. Yet, during transcription, while RNA polymerase acts on the DNA template, there exists a region upstream of the RNA polymerase with hyper-negative supercoiling where R-loop formation can potentially occur (Dorman 2019).

Centromeric R-loops have been identified in different species, including fission yeast, maize, rice, *Arabidopsis*, and human (Fang et al. 2019; Kabeche et al. 2018; Xu et al. 2017). In particular, a genome-wide R-loop map of maize leaf has been generated by single-strand DNA ligation-based library construction from DNA-RNA hybrid immunoprecipitation by S9.6 antibody, followed by sequencing (ssDRIP-seq). R-loops are enriched in centromeric regions, especially in the binding regions of CENP-A/CenH3, and pericentric regions (Yang Liu et al. 2021).

In *S. pombe* pericentromeric outer repeats (*otr*), in addition to dsRNA (Folco et al. 2008), about half of the non-coding RNAs (ncRNAs) are associated with chromatin that shows sensitivity towards RNase H, which cleaves DNA-RNA hybrids (Nakama et al. 2012). These ncRNAs form DNA-RNA hybrids, bind to the RNA-induced transcriptional silencing (RITS) complex, and result in RNAi-driven platform for heterochromatin assembly (Nakama et al. 2012).

Some evidence also shows that R-loop accumulation can in fact help the maintenance of centromeric integrity. In human cells, R-loops have been detected at centromeres specifically during mitosis, and such presence of R-loop drives ATR signaling pathway activation, which is important for faithful chromosome segregation and genome stability (Kabeche et al. 2018). A recent study shows that inducing centromeric double-stranded breaks (DSBs) in G1 phase leads to an increase in centromeric H3K4me2 and transcription of human and mouse cenRNAs. CENP-A and HJURP interact with the deubiquitinase USP11, enabling formation of the RAD51–BRCA1–BRCA2 complex. This further facilitates the homologous recombination (HR) of DNA and RNA, despite the absence of a sister chromatid, forming DNA-RNA hybrids and causing DNA-end resection to facilitate the repair of DSBs (Yilmaz et al. 2021).

On the contrary, R-loop accumulation at centromere chromatin has been found to be detrimental for proper chromosome segregation and genomic stability (Allison & Wang 2019). In *Saccharomyces cerevisiae*, HPR1, which interacts with CENP-A/CSE4, prevents the accumulation of R-loop in the centromeric region. In *hpr1∆* mutant, a reduced level of CENP-A chaperone, SCM3, and a defective increased localization of histone H3 at the centromere have been observed (Mishra et al. 2021). Centromeric alpha-satellite array R-loops have been detected in human cancer cell lines. The presence of R-loop recruits BRCA1, which counteracts the accumulation of R-loops at the centromere, prevents centromere breakage, limits hyper-recombination, and ensures proper localization of CENP-A (Racca et al. 2021). Overexpression of pericenetric major satellite RNA transcription in mice sequesters BRCA1-associated network, causing destabilization of DNA replication forks, accumulation of R-loops, DNA damage, and induction of breast cancers (Zhu et al. 2018a, b). While the co-presence of centromeric R-loops and BRCA1 are evident in these studies (Yilmaz et al. 2021; Racca et al. 2021; Zhu et al. 2018a, b), the consequences appear to be different dependent on the situations. The above findings suggest that centromeric R-loops may have both positive and negative impacts on the centromere function and genome stability (Crossley et al. 2019). Further mechanistic understanding on how centromeric R-loop structure affects centromere function in different species or conditions will be useful to unveil its dynamic roles.

#### Centromeric circRNA

Circular RNAs (circRNAs) are formed by fusing the upstream 5′-splice site and the downstream 3′-splice site of a pre-messenger RNA (mRNA) through the process of back splicing (Li et al. 2018). CircRNAs are single-stranded, covalently closed RNA molecules, and are involved in RNA–protein complex formation and gene expression regulation (Li et al. 2018). CircRNAs have been explored in fly, worm, mouse, human, and plants, such as *Oryza sativa*, *Arabidopsis thaliana*, maize, and wheat (Memczak et al. 2013; Salzman et al. 2012; Westholm et al. 2014; Ye et al. 2015; Chen et al. 2018; Ye et al. 2015). However, due to the repetitive nature of centromeric DNA and the limitation of bioinformatics analysis tools to identify circRNAs, it has been difficult to identify centromeric circRNAs.

The first identified centromeric circRNAs are derived from centromeric retrotransposons in maize. These centromeric circRNAs bind to the centromere through R-loops (Liu et al. 2020). When using RNA interference to target against the sites of back-splicing in the circular cenRNAs, the level of circRNA, R-loops, and R-loop-induced chromatin loops decrease, and consequently, the level of CENP-A/CenH3 at centromere localization drops, indicating the significance of centromeric circRNA-derived R-loop in centromere integrity (Liu et al. 2020). The function and features of these centromeric circRNAs are just beginning to be understood.

### Functions of cenRNAs and pericenRNAs

#### Function of cenRNAs as a scaffold

Non-coding RNA may work as a scaffold that spatially organizes proteins. This kind of RNA–protein or RNA-RNA bindings can be part of the components of a soluble or chromatin-associated protein complex (Hentze et al. 2018). RNAs may play a role in soluble complex formation (Quénet & Dalal 2014). To identify RNA-dependent soluble complexes, a newly developed tool based on density gradient ultracentrifugation, called R-DeeP, has been used. The CENP-A chaperone, HJURP, has shown an RNA-dependent shift in size (Caudron-Herger et al. 2019). The HJURP complex shows an increase in the size of the protein complex upon RNase (RNase A, RNase I, RNase T1, RNase H, and RNase III) treatment. The authors attribute this increase of size to a gain in interaction partners due to the increased availability of binding sites when RNA is degraded (Caudron-Herger et al. 2019). Future research can determine whether it is the cenRNAs that play a role in such complex formation, and this will help to establish a more complete picture of the role of cenRNAs in soluble protein complex formation.

In addition to soluble protein complexes, transcribed RNAs can associate with proximal chromatin as evidenced by RNA–DNA proximity ligation approaches (Bell et al. 2018; Sridhar et al. 2017). In fact, well-known chromatin binding factors, such as Polycomb complex (Zhang et al. 2019) and CCCTC-binding factor (CTCF) (Hansen et al. 2019), not only bind to distinct DNA motifs but are also functionally associated with RNA. Polycomb complex (Davidovich et al. 2013) and CTCF (Hansen et al. 2019; Saldaña-Meyer et al. 2019) are abundant at the pericentric heterochromatin and interact with a RNA component that is critical to their functions.

#### Function of centromeric RNAs in inner centromere signaling

Inner centromere is the region in between the centromeric regions of the sister chromatids (Trivedi & Stukenberg 2016). The chromosomal passenger complex (CPC) accumulates at the inner centromere region (Trivedi & Stukenberg 2016). The CPC contains the mitotic kinase Aurora B, INCENP, survivin, and borealin (Trivedi & Stukenberg 2016), and can sense and respond to the pulling forces generated at the kinetochores (Bloom 2014). In some vertebrates, CPC components have been shown to pull down cenRNAs. For example, in *Xenopus*, Aurora B has been shown to bound to cenRNA, which regulates both Aurora B’s localization and its activation (Blower 2016). Shugoshin (SGO1), which protects centromeric cohesion from cleavage during prophase (McGuinness et al. 2005), has also been shown to associate with cenRNA in vitro (Liu et al. 2015). Potentially, this cenRNA-SGO1 interaction allows SGO1 to reach cohesin embedded in centromeric chromatin (Liu et al. 2015). However, how exactly cenRNAs affect the recruitment of SGO1 and CPC to facilitate mitotic progression are still not clear.

#### Functions of pericentric transcription and pericenRNAs in heterochromatin formation

As discussed in the above sections a1 and 3, the fission yeast *otr* pericenRNA transcripts are processed by the RNA interference pathway and are important for heterochromatin establishment (Folco et al. 2008). In mouse cells, H3K9 methyltransferases SUV39H1 and SUV39H2 associate with major satellite RNAs derived from pericentric regions (but not with minor satellite RNAs from the centromeric regions) (Camacho et al. 2017; Johnson et al. 2017). The protein-RNA interaction stabilizes their chromatin localization and facilitates H3K9 histone methylation and heterochromatin formation (Camacho et al. 2017; Johnson et al. 2017). In a chicken-human hybrid DT-40 cell line, in which the centromeres also contain unique DNA sequences (Krasikova et al. 2012), conditional knockout of Dicer has different effects on the pericentric regions and the core centromere (Fukagawa et al. 2004). RNAi-deficient cells cause defective localization of heterochromatin proteins, cohesin protein Rad21, and checkpoint protein BubR1, while the localization of centromere proteins, including CENP-A and CENP-C, are normal (Corless et al. 2020; Fukagawa et al. 2004; Johnson et al. 2017). Such result indicates the differences in the requirement of the RNA interference or Dicer pathway between these two regions (Fukagawa et al. 2004; Hall et al. 2002; Provost et al. 2002; Volpe et al. 2002).

#### Implications from centromeric and pericentric RNA misregulation and diseases

The association and consequences of cenRNA and pericenRNA upregulation with stresses, cancers, and diseases have been investigated and extensively reviewed (Arunkumar and Melters 2020; Hernández-Saavedra et al. 2017; Smurova & De Wulf 2018). Pericentric HSAT II are overexpressed in some cancers, which indicates its potential application in cancer diagnosis (Hall et al. 2017). Overexpression of pericentric satellite RNA can even induce breast cancer (Zhu et al. 2018b). In general, high levels of cenRNAs promote chromosomal instability (CIN), which correlates with tumor metastasis (Chan et al. 2017; Zhu et al. 2011). Understanding how different organisms fine tune the expression of cenRNAs and pericenRNAs and determining the relationship between cenRNA regulation in stressed and disease conditions will help to apply cenRNAs and pericenRNA as biomarkers for diagnosis or prognosis in cancers and other diseases (Arunkumar and Melters 2020; Smurova & De Wulf 2018). Modulation of cenRNA or pericenRNA levels could also be important for cancer prevention or treatment.

### Causes and functions of cenRNA transcript variants

#### Different start and end sites, and different cell cycle timings

**Fig. 2:**
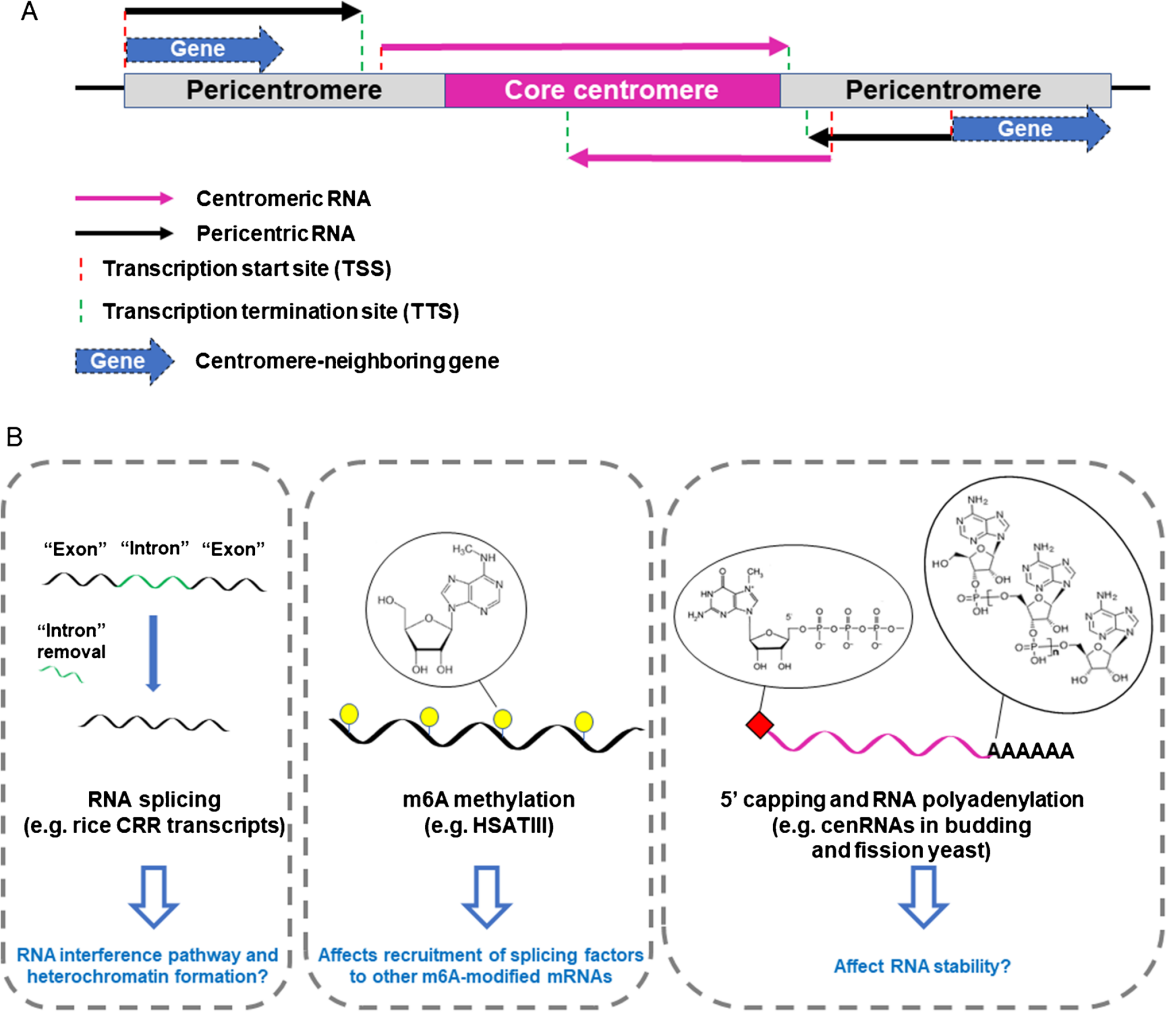
Mechanisms that contribute to cenRNA and pericenRNA variants and their potential functions. **A** RNA transcription could be initiated and terminated at different sites. CenRNA isoforms cover part or all of the core centromere regions, while pericenRNA isoforms are proximal to but do not cover the centromere regions. Pericentric RNAs may cover part of nearby genes, as observed in budding yeast (Hedouin et al. 2022). **B** CenRNAs and pericenRNAs could be processed by splicing (Neumann et al. 2007), m6A methylation (Xiao et al. 2016), 5′-capping (Choi et al. 2011), and polyadenylation (Arunkumar and Melters 2020; Choi et al. 2011; Ling & Yuen 2019; Neumann et al. 2007). The biological functions of these cenRNA processing processes and products are less certain, but could resemble those of mRNAs or sRNAs

The timing of centromeric transcription vary among organisms, but appears to correlate with the timing of CENP-A loading, as discussed briefly in b1 above and more extensively reviewed previously (Arunkumar and Melters 2020; Perea-Resa et al. 2018). Yet, within the same organism, cenRNA variants may be observed from different stages of the cell cycle, as evidenced by isoform RNA sequencing using long-read sequencing techniques in G1 and S phase in *S. cerevisiae* (Hedouin et al. 2022). The most abundant transcription isoform of each chromosome can be identified and classified based on the TSS: they can be initiated at nearby gene promoter or terminator sites, from anti-sense initiation of a neighboring gene, transcription readthrough from a neighboring gene, or initiation in an intergenic region (Fig. 2A) (Hedouin et al. 2022). However, they usually end upstream of the centromere in G1 (Hedouin et al. 2022; Ling & Yuen 2019). Only in S phase, the transcription leakage through into CDEI or CDEIII, due to centromere DNA replication and removal of CBF1 from CDEI, leading to the generation of cenRNAs (Hedouin et al. 2022). Even so, most identified cenRNAs do not encompass the full centromere (all three CDEs) (Hedouin et al. 2022). By 5′ and 3′ rapid amplification of cDNA ends (RACE), multiple *S. cerevisiae* centromeric transcript variants with different lengths (462–1754 nt) derived from both sense and anti-sense strands from three chromosomes have been characterized (Ling & Yuen 2019), consistent with the isoform RNA sequencing (Hedouin et al. 2022).

#### Different stabilities

In human cells, cenRNAs derived from different alpha-satellite arrays show different stability (Arunkumar and Melters 2020). CenRNA alpha-satellite generated within “active centromere” arrays (e.g., DXZ1 or D17Z1) associate with CENP-A and CENP-C, and are more stable compared to the transcripts from “inactive pericentric” arrays (e.g., D17Z1-B), which associate with CENP-B (McNulty et al. 2017).

#### Processing into shorter cenRNAs

Only a few studies have unveiled the details about cenRNA processing, possibly because it is difficult to trace all cenRNA variants, which may be short-lived or unstable. In mouse cells, while the core centromere precursor transcripts are about 2–4 kb, 120 nt of minor satellite transcripts are also identified (Bouzinba-Segard et al. 2006; Ferri et al. 2009). These data indicate that after being transcribed as a RNA precursor, cenRNA might be processed, by alternative splicing and post-transcriptional modifications (Wilkinson et al. 2020). In rice, > 3-kb transcripts derived from centromere retrotransposon (CRR) arrays have been found to be processed into small RNA (sRNA) of about 24 nt in length, which plays critical roles in the RNAi pathway to maintain heterochromatin (Neumann et al. 2007). In addition, splicing of intron-like elements in pericentric RNAs in fission yeast has been observed. Mutation of a splicing factor, PRP16, leads to pericentric heterochromatin defects (Vijayakumari et al. 2019).

#### m6A methylation and splicing

Recently, researchers have explored the impact of pericenRNA levels on mRNA splicing (Ninomiya et al. 2021; Vourc’h et al. 2022). Many target mRNAs contain N(6)-methyladenosine (m6A). m6A modification recruits a m6A reader, YTHDC1, to promote splicing (Xiao et al. 2016). Upon thermal stress, HSATIII RNAs, which are generated from pericentromeric regions, are highly expressed in HeLa cells. This might sequester the m6A writer complex and YTHDC1 in nuclear stress bodies (nSB) (Fig. 2B). This process will diminish m6A methylation and YTHDC1 binding to the target mRNAs, which in turn repress the splicing of target mRNAs (Ninomiya et al. 2021).

#### Polyadenylation

Telomeric repeat-containing RNA (TERRA) is a lncRNA generated from telomere DNA. About 7% of human TERRA is polyadenylated, whereas most of the yeast TERRA is polyadenylated (Feuerhahn et al. 2010). CenRNA polyadenylation has also been found in different organisms, including budding and fission yeast, rice, mice, and humans (Arunkumar and Melters 2020; Choi et al. 2011; Ling & Yuen 2019; Neumann et al. 2007). In budding yeast, centromeric transcripts with polyadenylated tails have been detected by 3′ RACE with the oligo (dT) primer (Ling & Yuen 2019). Similarly, various poly (A) tails with different termination sites were identified in rice by 3′ RACE (Fig. 2B) (Neumann et al. 2007). However, it is possible that not all cenRNA and pericenRNAs are polyadenylated. Similarly, 5′ capping of cenRNAs or pericenRNAs has also been reported in fission yeast (Arunkumar and Melters 2020; Choi et al. 2011). However, the percentage of 5′ capping or 3′ polyadenylated cenRNA and pericenRNAs might also vary among different species, and whether polyadenylation of RNA Pol II-derived centromeric transcripts contributes only to their stability or has additional functions needs further exploration.

In summary, many cenRNA and pericenRNA variants are generated due to multiple processes during and after transcription, including different transcription start and termination sites, alternative splicing, and modifications. Whether the cells need to have a repertoire of cenRNA variants in different abundancies remains unclear. Some cenRNAs could be more highly expressed only because of the leaky nearby gene expression. Studying different transcriptional variants will be useful to determine whether cenRNAs function interchangeably, potentially *in trans*, or whether individual cenRNA variants have unique functions, possibly *in cis*.

## Future perspectives

Many previous studies analyzed centromere transcription and cenRNAs used population-based assays, including reverse transcription-quantitative polymerase chain reaction (RT-qPCR) and RNA short-read sequencing (RNA-seq). The average behaviors of cenRNAs across a cell population may not allow accurate characterization of the nature, number, and variants of cenRNAs in individual cells, especially many cenRNAs are lowly expressed and induced only in specific cell cycle timing (Biscotti et al. 2015; Blower 2016; Fachinetti et al. 2013; McNulty et al. 2017; Quénet & Dalal 2014). Knowledge on centromeric RNA has been increasing very quickly with the advancing single-molecule long-read and modification sequencing techniques. Microscopy approaches to visualize cenRNA transcripts in individual cells may help to resolve this averaging effect problem. These single-cell techniques may include detecting cenRNAs by LNA probes in mitotic chromosome spreads, RNA-fluorescence in-situ hybridization combined with immunofluorescence (RNA-FISH-IF) (Kochan et al. 2015, aptamer-tagged non-coding RNA (Autour et al. 2018), and single-molecule fluorescence in-situ hybridization (smFISH) (Raj et al. 2008; Rošić et al. 2014), a strategy that has been used to detect unique mRNAs and long non-coding RNAs (lncRNAs).

Experiments that affect the transcription process will unavoidably affect the corresponding transcript level as well. Directly comparing the centromere, CIN or cell cycle phenotypes by manipulating centromeric transcription and just the cenRNA level can further elucidate the function of each component. The additional phenotypes changes from manipulating transcription could be attributed to the function of the act of transcription, but of course perturbation of transcription has pleiotropic effects that have to be taken into account. Alternatively, manipulation of a particular transcription stage or RNA processing event may unveil the importance of each step. Exploring the characteristics and mechanism of cenRNA in more diverse model organisms will also provide a wider perspective for a potential conserved function.

## Data Availability

Not applicable.
